# Association of interferon-based therapy with risk of autoimmune diseases in patients with chronic hepatitis C virus infection: A population-based Taiwanese cohort study

**DOI:** 10.3389/fimmu.2022.992819

**Published:** 2022-10-07

**Authors:** Shu-Ming Chou, Hsing-Jung Yeh, Tzu-Min Lin, Yu-Sheng Chang, Hui-Ching Hsu, Yu-Chuan Shen, Tzu-Tung Kuo, Jin-Hua Chen, Shu-Chuan Chen, Chi-Ching Chang

**Affiliations:** ^1^ Department of Internal Medicine, Taipei Medical University Hospital, Taipei, Taiwan; ^2^ Division of Gastroenterology and Hepatology, Department of Internal Medicine, Taipei Medical University Hospital, Taipei, Taiwan; ^3^ Division of Gastroenterology and Hepatology, Department of Internal Medicine, School of Medicine, College of Medicine, Taipei Medical University, Taipei, Taiwan; ^4^ Division of allergy, immunology and Rheumatology, Department of Internal Medicine, School of Medicine, College of Medicine, Taipei Medical University, Taipei, Taiwan; ^5^ Division of Allergy, Immunology and Rheumatology, Department of Internal Medicine, Taipei Medical University Hospital, Taipei, Taiwan; ^6^ Division of Allergy, Immunology, and Rheumatology, Department of Internal Medicine, Shuang Ho Hospital, Taipei Medical University, New Taipei City, Taiwan; ^7^ Division of Allergy, Immunology and Rheumatology, Department of Internal Medicine, Wan Fang Hospital, Taipei Medical University, Taipei, Taiwan; ^8^ Biostatistics Center, College of Management, Taipei Medical University, Taipei, Taiwan; ^9^ Graduate Institute of Data Science, College of Management, Taipei Medical University, Taipei, Taiwan; ^10^ Department of Mathematics and Statistics, Idaho State University, Pocatello, ID, United States

**Keywords:** hepatitis C virus, interferon-based therapy, autoimmune disease, Grave's disease, Hashimoto thyroiditis

## Abstract

**Background:**

Interferon in combination with ribavirin has been the standard of care for chronic hepatitis C virus infection (HCV) for the past few decades. However, its effect on the risk of autoimmune diseases (ADs) among patients with HCV infection remains unclear. We assessed the potential association between interferon-based therapy (IBT) and AD risk in patients with HCV infection.

**Methods:**

This retrospective cohort study identified patients diagnosed with HCV infection between January 1, 2006, and December 31, 2015, from Taiwan’s National Health Insurance Research Database. In total, 16,029 patients with HCV infection who received IBT and 141,214 patients with HCV infection who did not receive IBT were included. Both cohorts were followed up to assess the development of ADs. Hazard ratios (HRs) were calculated using the Cox proportional hazards regression model, which was adjusted for potential confounders.

**Results:**

The median follow-up period for IBT and non-IBT users was 4.53 and 3.34 years, respectively. No significant difference in the risk of overall ADs (adjusted HR [aHR]: 0.96, 95% confidence interval [CI]: 0.81–1.14) or systemic ADs (aHR: 0.88, 95% CI: 0.71–1.10) was noted during the study period. However, a slight increase in the risk of organ-specific ADs was noted among IBT users (incidence rate ratio: 1.33, 95% CI: 1.02–1.72). Furthermore, analysis of AD subgroups revealed a significant increase in the risks of Graves’ disease (aHR: 6.06, 95% CI: 1.27–28.8) and Hashimoto’s thyroiditis (aHR 1.49, 95% CI 1.01–2.21) among IBT users.

**Conclusions:**

IBT use increases the risk of autoimmune thyroid diseases (Hashimoto’s thyroiditis and Graves’ disease) in patients with HCV infection to a greater extent than non-IBT use.

## Introduction

Hepatitis C virus (HCV) is the causative agent of a type of hepatitis, previously known as non-A, non-B hepatitis (1). More than 71 million people worldwide are chronically infected with HCV in 2015 (2). According to Taiwan’s National Health Insurance Research Database (NHIRD), 400,000 hepatitis C carriers existed in Taiwan till 2015. HCV infection predisposes patients to hepatocellular carcinoma, liver failure, and liver cirrhosis (3).

Chronic HCV infection can trigger an immune response in the host. Thus, HCV infection is associated with numerous extrahepatic disorders (4), such as type 2 mixed cryoglobulinemia and B-cell lymphoma. Agnello et al. (5) found at least 36 extrahepatic disease manifestations, mainly autoimmune disorders such as Sjogren’s syndrome, systemic lupus erythematosus, autoimmune hemolytic anemia, antiphospholipid syndrome, autoimmune hemolytic anemia, Behcet’s syndrome, autoimmune thyroiditis and dermatomyositis have been reported to be associated with HCV infection. Sayiner et al. also reported 2%–38% of patients with HCV infection have manifestations of rheumatological features and associated with many autoimmune rheumatic disorders, such as rheumatoid arthritis (RA) (6).

Type I interferons (IFNs) are cytokines and they exhibit pleiotropic effects, such as the induction of inhibition of cell growth, regulation of apoptosis, and cell-autonomous antiviral resistance. Moreover, type I IFNs can regulate immune effector functions and act as signals linking innate and adaptive immune responses (7). IFN-α has become the cornerstone of antiviral therapy for HCV infection since the 1980s. After the completion of antiviral treatment, Pegylated IFNs can lead to a significant increase in a sustained virologic response (8). Although direct-acting antiviral agents are now becoming a popular and successful therapeutic option, the effect of IFN-based therapy (IBT) still needs to be investigated. The occurrence of IFN-α-associated autoimmunity has been reported to range from 4% to 19% (9, 10). Furthermore, autoimmune disorders, such as systemic lupus erythematosus (SLE), RA, polymyositis psoriatic arthropathy, sarcoidosis, autoimmune hemolysis, autoimmune thyroid disease, and immune thrombocytopenia, may occur during IFN-α therapy (8, 11).

Few studies have assessed the effect of IBT on the risk of autoimmune diseases (ADs) in patients with HCV infection and it is impossible to conduct a randomized clinical trial to know the effect of IBT. Hence, we used reimbursement claims data from NHIRD to examine the association between IBT for HCV infection and the risk of ADs.

## Materials and methods

### Data sources

NHI program which covers more than 99% of Taiwan’s population was launched in 1995 by the Taiwanese government (12). The NHRI maintains and updates the NHIRD, which contains the registration files and claims data of the beneficiaries of the NHI program. After the NHRI approved this study, we were able to assess the data of patients using scrambled patient identification numbers. In this dataset, the diagnostic codes were based on the International Classification of Diseases, Ninth Revision, Clinical Modification (ICD-9-CM), and the diagnoses were performed in accordance with the approved guidelines. Our study was approved by the Taipei Medical University Institutional Review Board (Approval Number N201908055). Informed consent was not required due to the dataset contained deidentified secondary data only for research purposes.

### Study design and participants

This retrospective cohort study was conducted using data from the NHIRD. To ensure the validity and reliability of diagnoses, only adult patients who received HCV infection diagnoses (ICD-9-CM codes 070.41, 070.44, 070.51, 070.54, and V02.62) that were confirmed by in an inpatient setting or three or more ambulatory care claims. Patients who (1) were diagnosed with HBV infection (2), were younger than 18 years and had unknown age or sex (3), were diagnosed with ADs before the index date (4), were diagnosed with ADs within 6 months after the index date (5), had a follow-up duration of less than 6 months (6), were diagnosed with any form of cancer within 1 year before the cohort entry date, and (7) received IBT for less than 16 weeks were excluded from the study. The index date was the first date of receiving IBT for the treated cohort. The patients who never received IBT were consisted of untreated cohort during the study period. Patients were followed up from the entry date to the development of loss to follow-up, death, ADs or the end of the study.

### Interferon-based therapy exposure

Six months IBT for all HCV genotypes has been reimbursed by the NHI Administration since October 1, 2 003. A combination of IBT and ribavirin is most prescribed among patients with HCV infection (97.8%) (13). The IBT regimen in our study consisted of a combination of pegylated IFN-α-2b (including the non-pegylated form) and ribavirin according to Anatomical Therapeutic codes. The duration of antiviral therapy ranged from 24 to 48 weeks.

### Outcome measurement and comorbidities

Enrolled patients were observed up until occurrence of the interest outcomes and the end of the study. They were followed up for AD outcomes included systemic ADs (many different organs and tissues is targeted by immune system)and organ-specific ADs(a particular organ or tissue is targeted by immune system): Patients with systemic ADs were identified by Registry for Catastrophic Illness Patient Database (RCIPD) for the following diseases: SLE (ICD-9-CM code 710.0); RA (ICD-9-CM code 714.0); SSc (ICD-9-CM code 710.1); primary SjS (ICD-9-CM code 710.2); PM/DM (ICD-9-CM code 710.4/710.3); and Takayasu arteritis (ICD-9-CM code 446.7), temporal arteritis (ICD-9-CM code 446.5), polyarteritis nodosa (ICD-9-CM code 446.0), myasthenia gravis (ICD-9-CM code 358.0), and IBD (ICD-9-CM code 555.9). Takayasu arteritis, temporal arteritis, and polyarteritis nodosa are types of systemic vasculitis. In addition, we excluded patients with comorbidities such as systemic lupus erythematous, rheumatoid arthritis, scleroderma, polymyositis, dermatomyositis and HCV infection to limit our study sample to pSS. The following organ-specific ADs without catastrophic illness certification were identified using ICD-9-CM codes that appeared once in the discharge diagnosis for hospitalized patients or thrice within a year in outpatient diagnoses: ankylosing spondylitis (ICD-9-CM code 720.0), and psoriasis (ICD-9-CM code 696), type 1 diabetes mellitus (ICD-9-CM code 250.01), autoimmune hemolytic anemia (ICD-9-CM code 283.0), Addison’s disease (ICD-9-CM code 255), Graves’ disease (ICD-9-CM code 242.0), Henoch-Schönlein purpura (ICD-9-CM code 287.0),Hashimoto’s thyroiditis (ICD-9-CM code 245.2).

For each patient, comorbidities were assessed using the Charlson comorbidity index (CCI) score. The CCI categorizes comorbidities based on the ICD diagnosis codes found in administrative data, such as hospital abstract data.

### Statistical analysis

The incidence rates of ADs were estimated during the follow-up period in patients who received pegylated IFN α-2b and in those who did not. In addition, the incidence rate ratio (IRR) was calculated to assess the unadjusted risk of AD occurrence in the two groups. The confidence intervals (CIs) of IRRs were calculated using Poisson distribution and test-based methods. We used Cox proportional hazards regression model to estimate the adjusted hazard ratios (aHRs) and 95% CIs. In the Cox regression model, the aHRs were adjusted for sex, age, and comorbidities. We used the Kaplan–Meier estimator to assess the cumulative incidence of overall ADs, organ-specific ADs, and systemic ADs, and *p* < 0.05 was considered statistically significant. We used Student’s *t* test and Pearson’s chi-squared test, respectively to analyze the baseline characteristics, differences in continuous and categorical variables between the groups. We considered the all results of statistical analyses are significant at *p* < 0.05. SAS 9.4 and R 3.6.3 were used for the analyses.

## Results

### Baseline characteristics of the IBT and non-IBT groups

**Figure 1:**
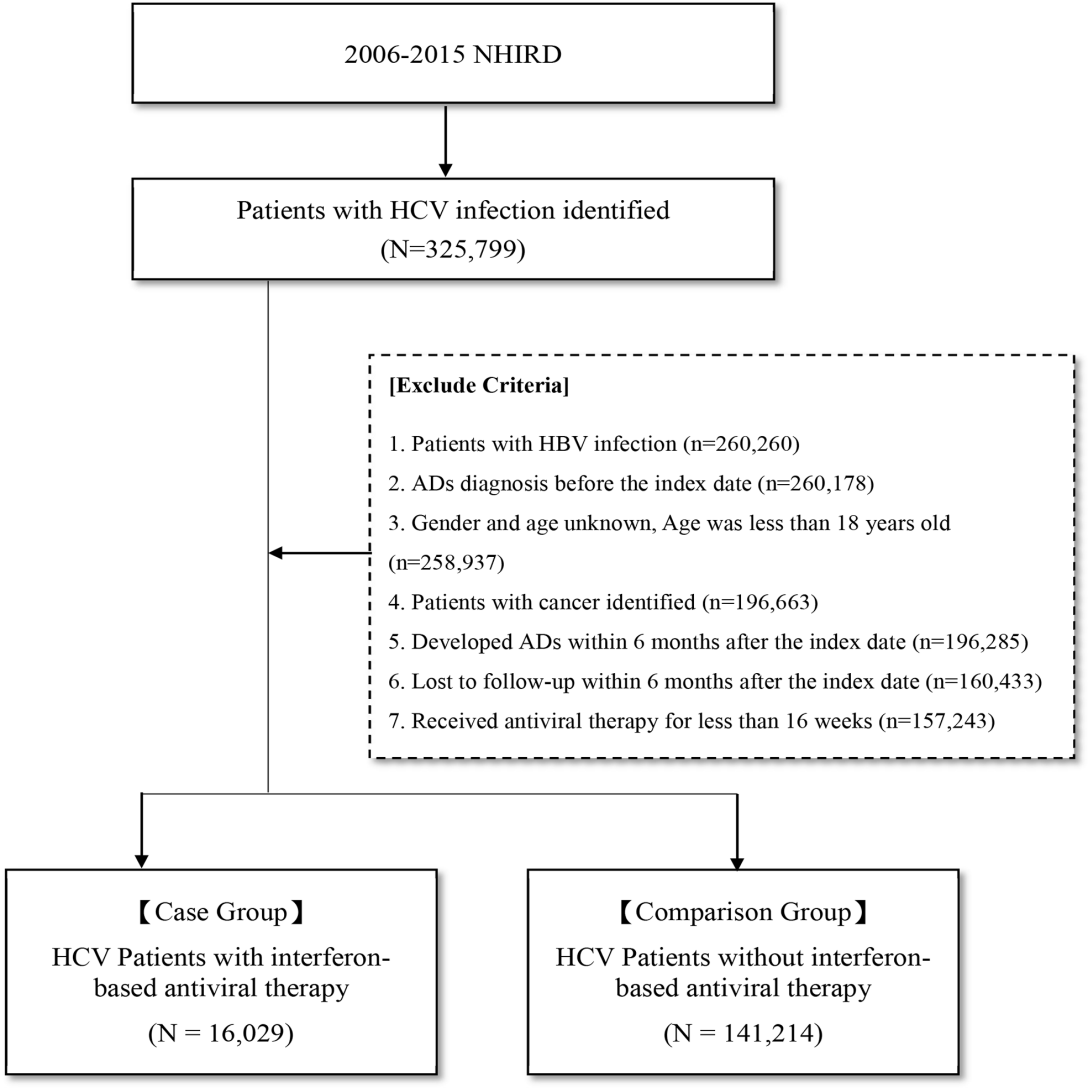
Flow Chart for Study Design. AD, autoimmune disease; HCV, hepatitis C virus; NHIRD, National Health Insurance Research Database.

As shown in **Figure 1**, in this study, 325,799 patients diagnosed with HCV infection between January 1, 2006, and December 31, 2015, were identified. Patients who (1) were diagnosed with HBV infection (n = 260,260) (2), were diagnosed with AD before the index date (n = 260,178) (3), had unknown sex or age or were aged less than 18 years (n = 258,937) (4), had cancer (n = 196,663) (5), were diagnosed with ADs within 6 months after the index date (n = 196,285) (6), were lost to follow-up within 6 months after the index date (n = 160,433), and (7) received antiviral therapy for less than 16 weeks (n = 157,243) were excluded from this study. After excluding these patients, a total of 16,029 patients with HCV infection who received IBT were included in the IBT group (case group), and 141,214 patients with HCV infection who did not receive IBT were included in the non-IBT group (comparison or control group). The non-IBT group had higher mean CCI scores and a higher number of male patients than the IBT group (**Table 1**).

**Table 1:** Baseline Characteristics of IBT and Non-IBT Group.

	IBT group (N = 16029)	Non-IBT group (N = 141214)	P-Value
**Gender**			<.0001
**Female**	7784 (48.56%)	71692 (50.77%)	
**Male**	8245 (51.44%)	69522 (49.23%)	
**Age Group**			<.0001
**18-30**	540 (3.37%)	5493 (3.25%)	
**31-40**	1782 (11.12%)	14338 (10.15%)	
**41-50**	3452 (21.54%)	23520 (16.66%)	
**51-60**	5708 (35.61%)	34864 (24.69%)	
**61-70**	3659 (22.83%)	30353 (21.49%)	
**71-80**	857 (5.35%)	23776 (16.84%)	
**> 80**	31 (0.19%)	9770 (6.92%)	
**Mean (SD)**	53.48 (11.51)	58.43 (15.02)	<.0001
**Median (IQR)**	55 (15)	59 (22)	<.0001
**CCI Score**			<.0001
**0**	3201 (19.97%)	61495 (43.55%)	
**1**	8273 (51.61%)	46756 (33.11%)	
**2**	3318 (20.70%)	20146 (14.27%)	
**3**	744 (4.64%)	6875 (4.87%)	
**>=4**	493 (3.08%)	5942 (4.21%)	
**Mean (SD)**	1.23 (1.08)	0.98 (1.24)	<.0001
**Median (IQR)**	1 (1)	1 (1)	<.0001
**Follow up time**			
**Mean (SD)**	4.53 (2.40)	3.34 (2.31)	<.0001
**Median (IQR)**	4.41 (3.39)	2.79 (3.48)	<.0001

**CCI**, Charlson comorbidity index; IBT, interferon-based therapy.

### IRR and HR of the risk of ADs between the IBT and non-IBT groups

**Table 2** presents the incidence of ADs in the two groups. During the study period, no significant difference in the risk of overall ADs (IRR: 1.10, 95% CI: 0.93–1.29; aHR: 0.96, 95% CI: 0.81–1.14) or systemic ADs (IRR: 0.99, 95% CI: 0.80–1.21; aHR: 0.88, 95% CI: 0.71–1.10) was noted between the groups. By contrast, the IBT group had a higher risk of organ-specific ADs (IRR: 1.33, 0.99, 95% CI: 1.02–1.72).

**Table 2:** Incidence Rate Ratio and Hazard Rate of the Risk of ADs between IBT and non-IBT group.

	Event	Person Year	IncidenceRate†	IRR		aHR	
Overall ADs					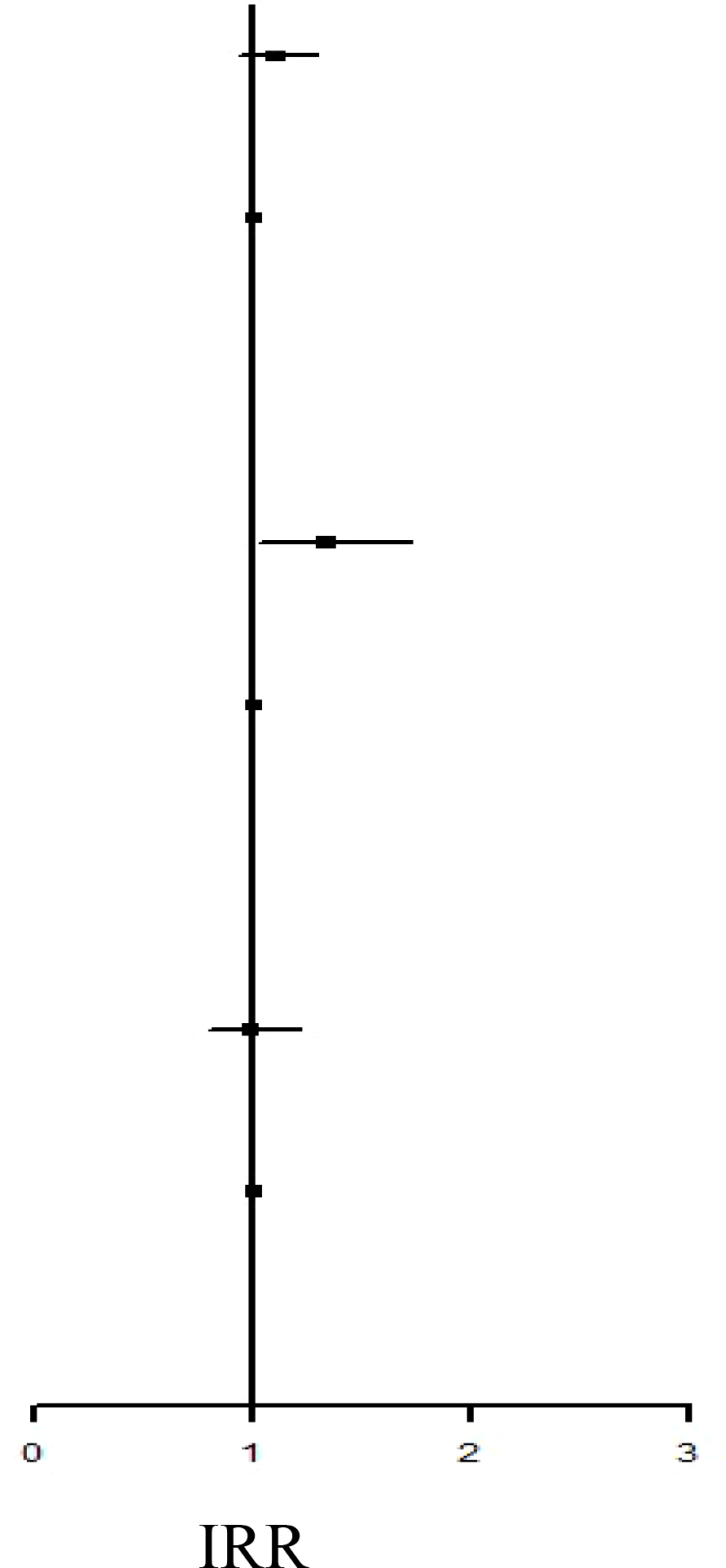		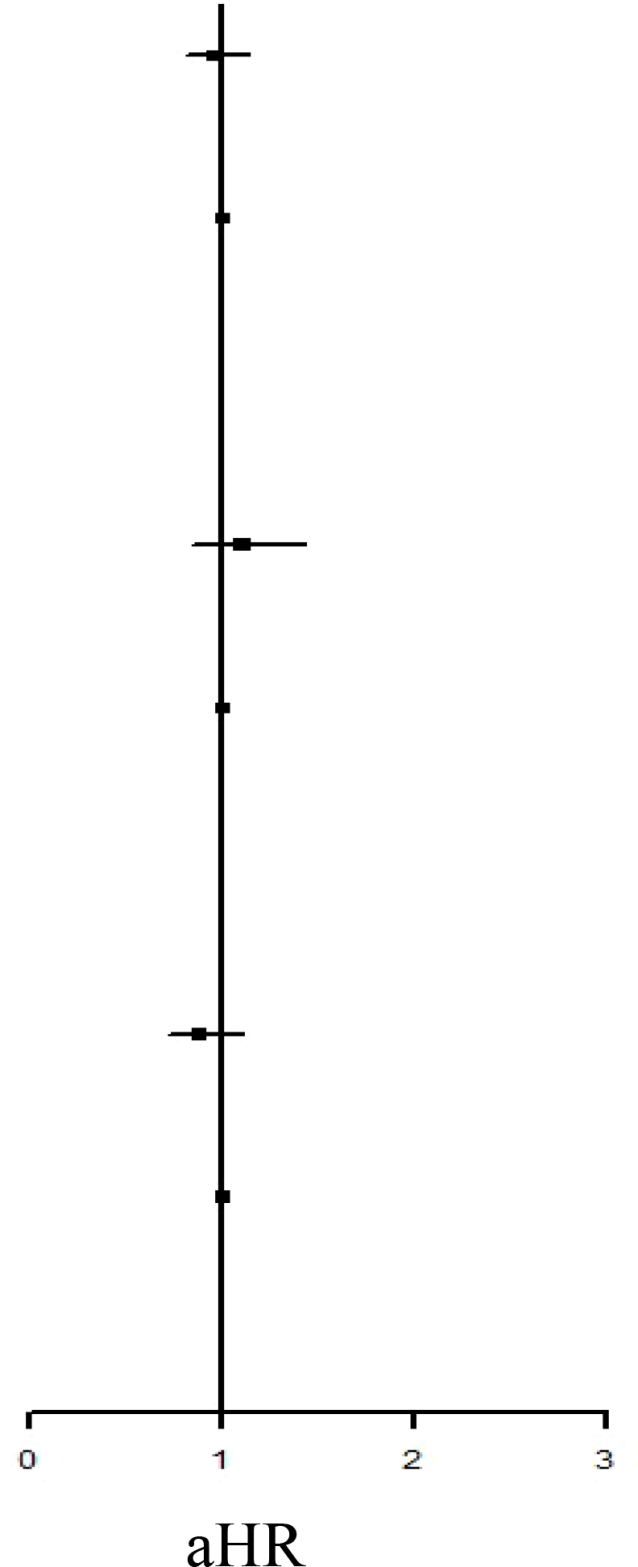
IBT Group	169	72538.54	232.98	1.10 (0.93-1.29)		0.96 (0.81-1.14)	
Non-IBT Group	1000	472137.61	211.80	Ref.		Ref.	
Organ-Specified ADs							
IBT Group	68	72799.57	93.41	1.33 (1.02-1.72)*		1.10 (0.84-1.43)	
Non-IBT Group	333	473678.61	70.30	Ref.		Ref.	
Systemic ADs							
IBT Group	102	72746.42	140.21	0.99 (0.80-1.21)		0.88 (0.71-1.10)	
Non-IBT Group	673	472902.14	142.31	Ref.		Ref.	

IR, incidence rate was incidences of per 100,000 person-years. HR, hazard ratio; aHR, adjusted hazard ratio; CI, confidence interval.

*P <0.05.

### IRR and HR of the risk of AD subtypes between the IBT and non-IBT groups

Regarding organ-specific ADs, the incidence rates of Graves’ disease (IRR: 8.67, 95% CI: 1.94–38.7; aHR: 8.67, 95% CI: 1.94–38.7) and Hashimoto’s thyroiditis (IRR: 1.71, 95% CI: 1.17–2.50; aHR: 1.49, 95% CI: 1.01–2.21) were significantly higher in the IBT group than in the non-IBT group (**Table 3**). No significant difference was noted in the incidence of systemic ADs between the two groups (**Table 4**).

**Table 3:** Incidence Rate Ratio and Hazard Rate of the Risk of subgroup ADs between IBT and non-IBT group.

	Event	Person Year	IncidenceRate†	IRR		aHR	
Addison’s disease					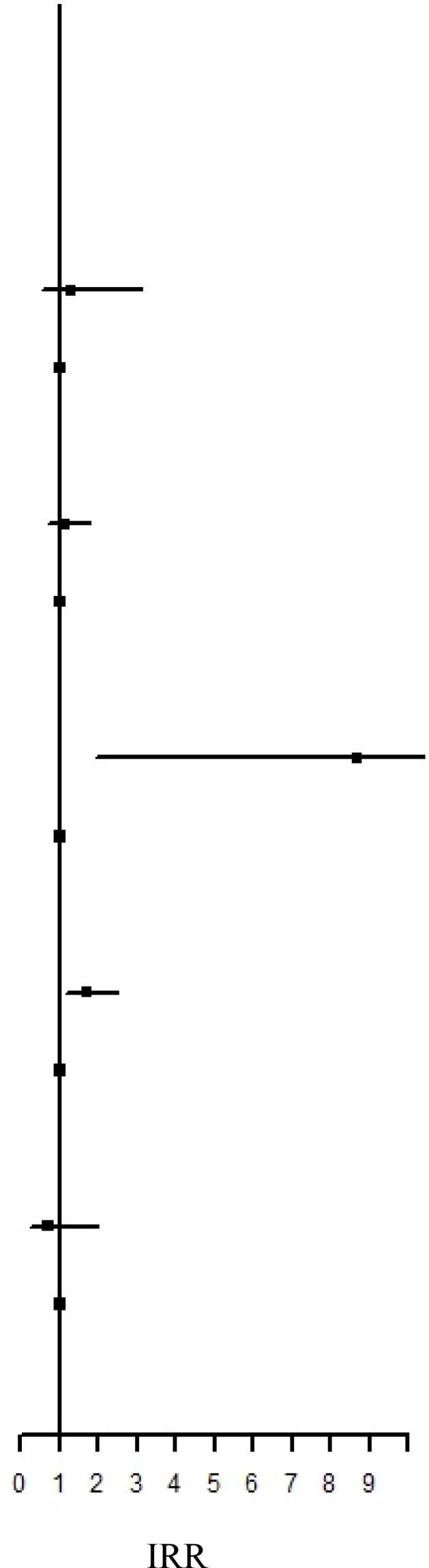		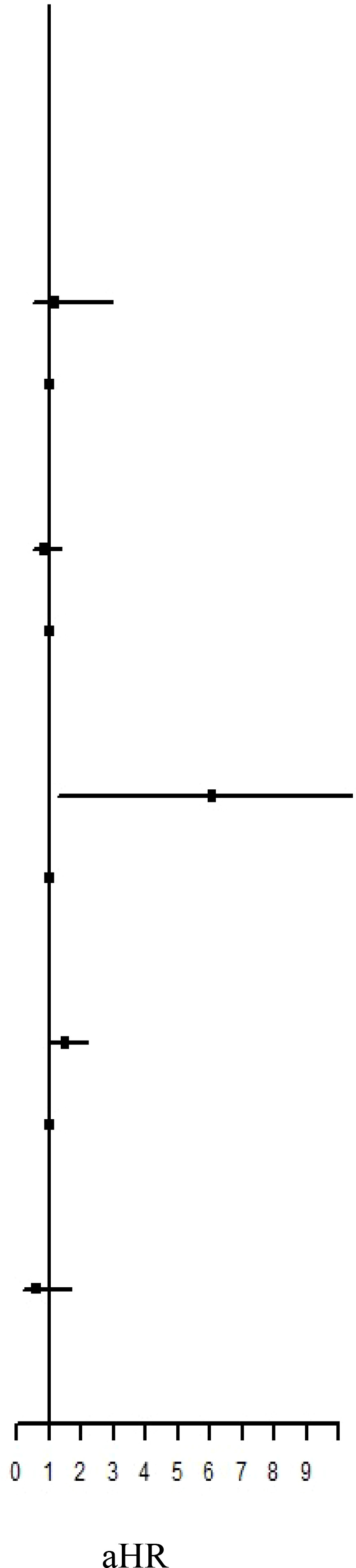
IBT Group	0	73009.17	0.00	N.A		N.A	
Non-IBT Group	1	474449.96	0.21	Ref.		Ref.	
Autoimmune hemolytic anemia							
IBT Group	6	72991.22	8.22	1.30 (0.54-3.12)		1.19 (0.47-2.96)	
Non-IBT Group	30	474390.59	6.32	Ref.		Ref.	
DM Type I							
IBT Group	19	72948.69	26.05	1.10 (0.68-1.79)		0.83 (0.50-1.37)	
Non-IBT Group	112	474196.08	23.62	Ref.		Ref.	
Graves’ disease							
IBT Group	4	72999.83	5.48	8.67 (1.94-38.7)**		6.06 (1.27-28.8)*	
Non-IBT Group	3	474441.99	0.63	Ref.		Ref.	
Hashimoto’s thyroiditis							
IBT Group	34	72900.38	46.64	1.71 (1.17-2.50)**		1.49 (1.01-2.21)*	
Non-IBT Group	129	474111.38	27.21	Ref.		Ref.	
Henoch-Schonlein purpura							
IBT Group	4	73001.99	5.48	0.70 (0.25-1.97)		0.59 (0.20-1.67)	
Non-IBT Group	37	474381.40	7.80	Ref.		Ref.	
Immune thrombocytopenic purpura					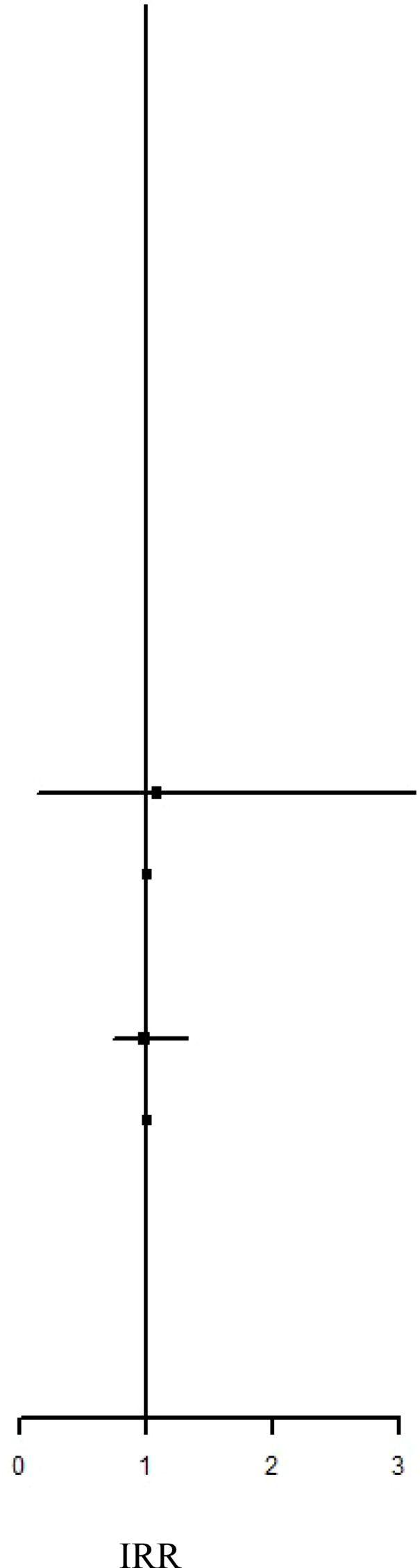		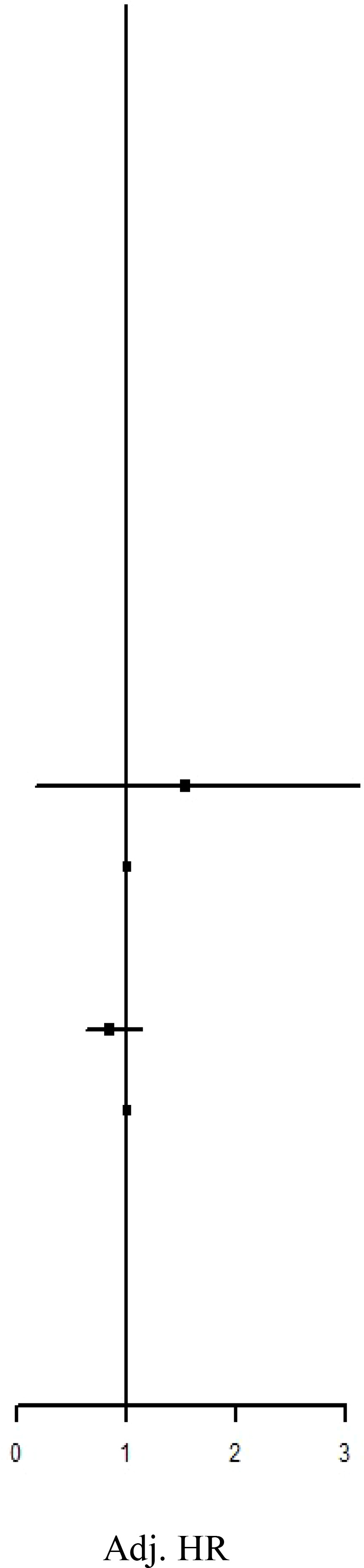
IBT Group	0	73009.17	0.00	N.A		N.A	
Non-IBT Group	0	474450.33	0.00	Ref.		Ref.	
Lupoid hepatitis							
IBT Group	0	73009.17	0.00	N.A		N.A	
Non-IBT Group	0	474450.33	0.00	Ref.		Ref.	
Myasthenia gravis							
IBT Group	0	73009.17	0.00	N.A		N.A	
Non-IBT Group	17	474413.93	3.58	Ref.		Ref.	
Inflammatory bowel disease							
IBT Group	1	73003.04	1.37	1.08 (0.13-8.99)		1.53 (0.17-13.7)	
Non-IBT Group	6	474439.44	1.26	Ref.		Ref.	
Ankylosing Spondylitis							
IBT Group	54	72886.06	74.09	0.98 (0.74-1.31)		0.84 (0.63-1.13)	
Non-IBT Group	357	473684.09	75.37	Ref.		Ref.	
Psoriasis							
IBT Group	0	73009.17	0.00	N.A		N.A	
Non-IBT Group	5	474435.43	1.05	Ref.		Ref.	

IR, incidence rate was incidences of per 100,000 person-years. HR, hazard ratio; aHR, adjusted hazard ratio; CI, confidence interval.

**P <0.01.

NA, not available.

**Table 4:** Incidence Rate Ratio and Hazard Ratio of the Risk of systemic ADs between IBT and non-IBT group.

	Event	Person Year	IncidenceRate†	IRR		aHR	
Polymyositis/dermatomyositis					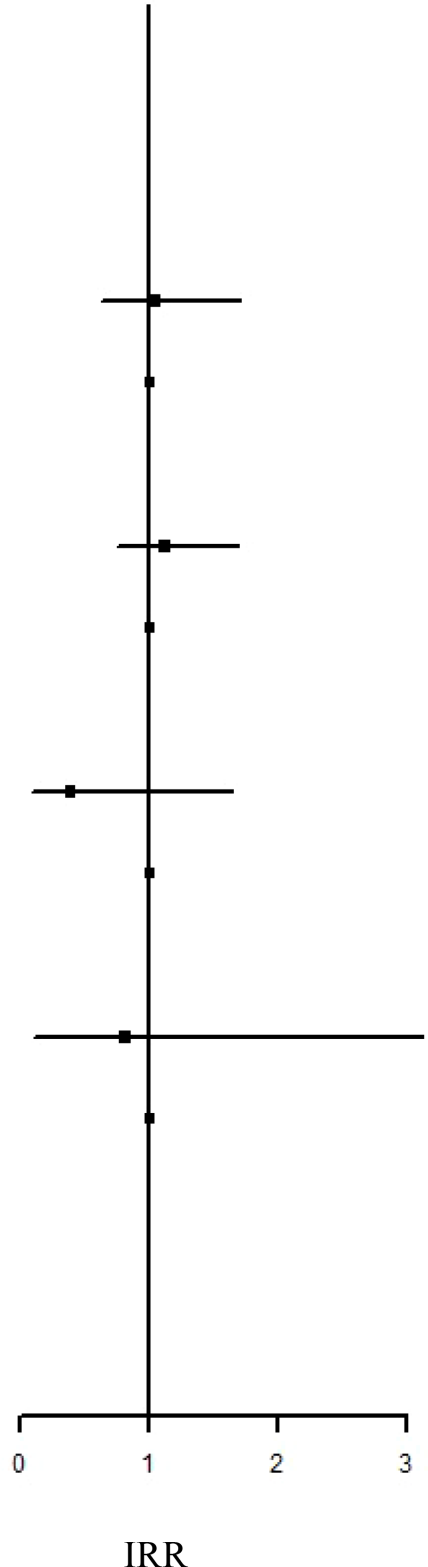		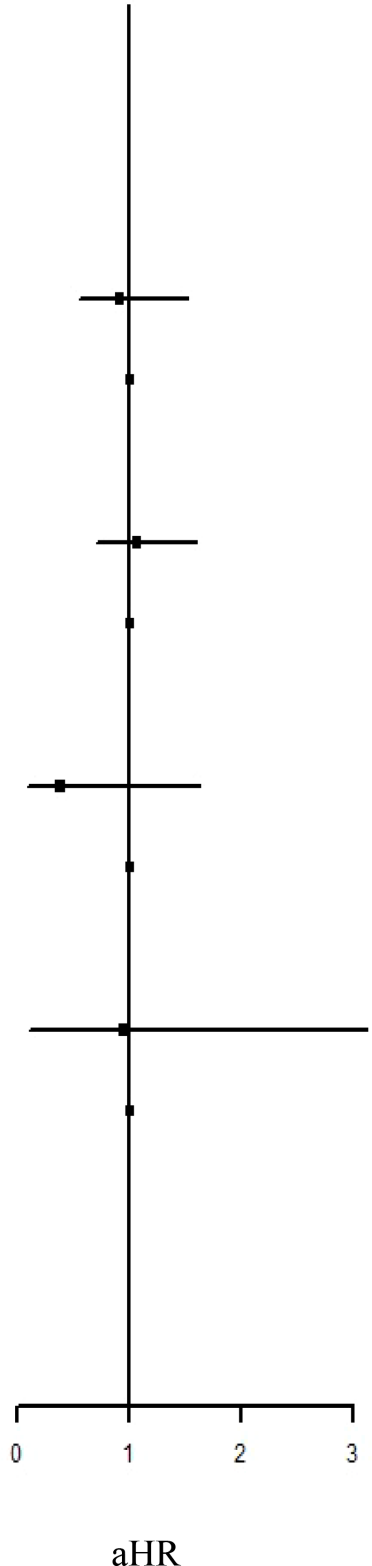
IBT Group	0	73009.17	0.00	N.A		N.A	
Non-IBT Group	7	474442.69	1.48	Ref.		Ref.	
Rheumatoid arthritis							
IBT Group	18	72946.54	24.68	1.04 (0.63-1.70)		0.91 (0.55-1.52)	
Non-IBT Group	113	474132.51	23.83	Ref.		Ref.	
Primary Sjogren’s syndrome							
IBT Group	28	72940.62	38.39	1.12 (0.75-1.68)		1.06 (0.70-1.60)	
Non-IBT Group	162	474072.65	34.17	Ref.		Ref.	
Systemic lupus erythematosus							
IBT Group	2	73000.58	2.74	0.39 (0.09-1.64)		0.38 (0.09-1.62)	
Non-IBT Group	33	474384.46	6.96	Ref.		Ref.	
Systemic sclerosis							
IBT Group	1	73006.74	1.37	0.81 (0.10-6.49)		0.94 (0.11-7.97)	
Non-IBT Group	8	474436.63	1.69	Ref.		Ref.	
Systemic vasculitis							
IBT Group	0	73009.17	0.00	N.A		N.A	
Non-IBT Group	1	474444.39	0.21	Ref.		Ref.	

IR, incidence rate was incidences of per 100,000 person-years. HR, hazard ratio; aHR, adjusted hazard ratio; CI, confidence interval; NA, not available.

### Comparison of cumulative incidence of organ-specific ADs between the IBT and non-IBT groups

**Figure 2:**
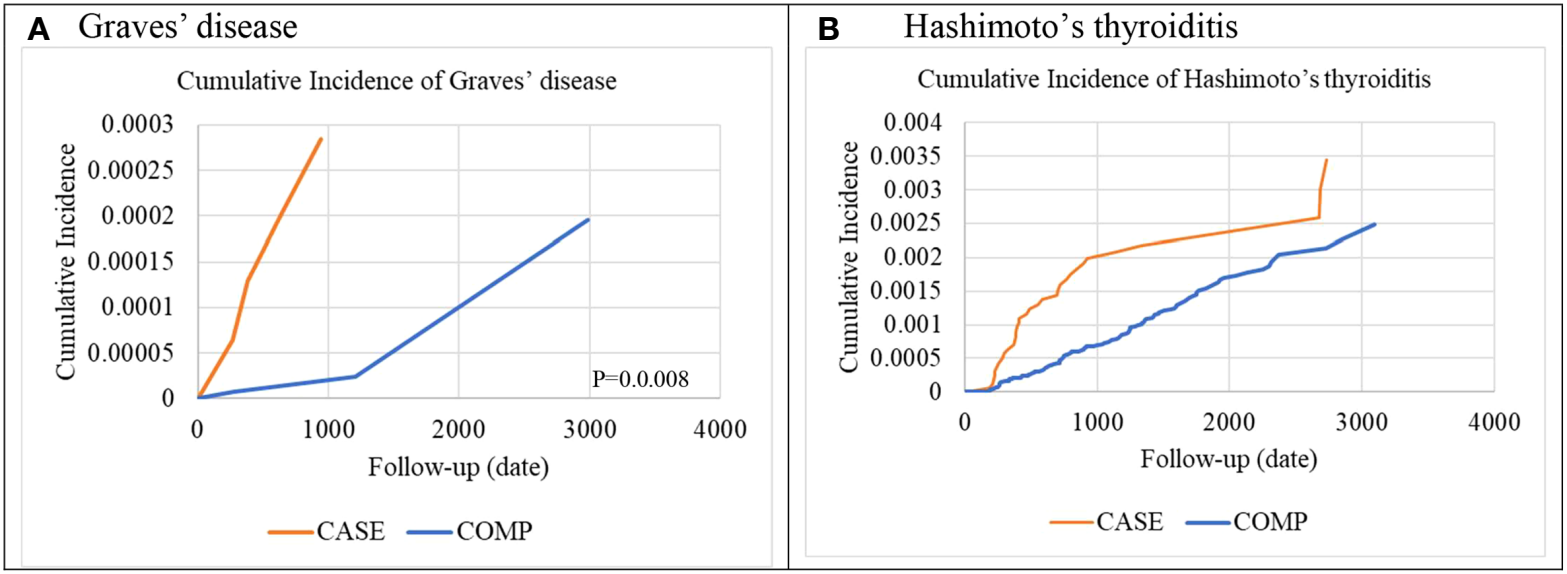
Cumulative incidence of organ-specific autoimmune diseases **(A)** Graves’ disease **(B)** Hashimoto’s thyroiditis.

A comparison of the cumulative incidence of organ-specific ADs between the IBT and non-IBT groups is presented in **Figure 2**. The Kaplan–Meier estimates of organ-specific AD–free survival revealed a significantly higher incidence rate of organ-specific ADs in the IBT group than in the non-IBT group (Graves’ disease, **Figure 2A**; Hashimoto’s thyroiditis, **Figure 2B**).

## Discussion

To the best of our knowledge, this is the largest study to assess the association between IBT and the risk of ADs in patients with HCV infection. Our findings revealed that patients with HCV infection who received IBT did not have an increased risk of overall ADs or systemic ADs but had a slightly increased risk of organ-specific ADs. Furthermore, the IBT group had a significantly increased risk of autoimmune thyroid diseases (Graves’ disease and Hashimoto’s thyroiditis).

Chronic HCV infection can increase the risk of autoimmune thyroid diseases (14–16). However, our findings suggested that patients with HCV infection who were treated with IBT had a higher risk of autoimmune thyroid diseases than those who were not treated with IBT. A recent study using the NHIRD also reported a higher incidence rate of thyroid dysfunction among IFN-treated patients than among untreated patients with chronic HCV (17).

In a previous study, the incidence rate of thyroid disease was slightly higher than that of other ADs in patients receiving IBT (18). Among patients with HCV infection who were treated with IFNs, approximately 1.7% developed hypothyroidism and 0.6% developed hyperthyroidism (18). In another study, the incidence rate of thyroid disease after IBT was approximately 13.3% (19).

Tomer, Y. et al. reported that both immune-mediated and direct effects of IFN-α on thyroid function are involved in the mechanism of IFN-induced thyroiditis (20). Several pathways can underlie the immune-mediated effects of IFN-α. In particular, IFN-α receptor activation leads to the activation of the JAK–STAT pathway (21), resulting in the activation of numerous IFN-stimulated genes, including those encoding cytokines and adhesion molecules (19, 20). These effects can induce thyroid autoimmunity. IFN-α can increase the expression of major histocompatibility complex class I antigens on thyroid epithelial cells (22), which can lead to the activation of cytotoxic T cells and result in tissue damage and inflammatory response (23).

IFN-α can also shift the immune response to a T helper cell type 1 (Th1)-mediated pattern (24), leading to an increase in the production of IFN-γ and interleukin (IL)-2 (25). Ribavirin, an oral guanosine analog frequently used in combination with IFN to improve response and decrease relapse, may also alter the immune response (26). Moreover, IBT may result in autoimmune thyroiditis by enhancing the activity of lymphocytes, macrophages, and natural killer cells (23, 27–29); activating neutrophils and lymphocytes (20); inducing the release of IL-6 (23); and decreasing T-regulatory cell function by affecting the production of immunoglobulin (30, 31).

Hashimoto’s thyroiditis is a hypothyroidism-related disease. Although it is a Th1-mediated disease, it is also associated with the Th2 response. Hypothyroidism has been reported in patients with HCV infection (32). In a recent study assessing the thyroid function of patients with HCV infection who received IBT for 24–48 weeks, hypothyroidism was found to be the most frequent thyroid disease (33). Genetic and environmental factors play an important role in the occurrence of thyroid disease (19, 34).

Graves’ disease is a hyperthyroidism-related disease that is driven by the humoral immune response and Th2 cytokines. Although it is an antibody-mediated disease, it is predominantly a Th1-type cytokine disease (35). Therefore, Graves’ disease and Hashimoto’s thyroiditis have many common features. In both the diseases, the autoimmune response comprises both Th1 and Th2 types. Therefore, these diseases may be noted in patients with HCV infection who receive IBT.

Other ADs, such as RA, psoriasis, and polymyositis, have also been reported in patients with HCV infection who are treated with IFN-α (10, 36, 37). In addition, SLE is a frequently reported autoimmune rheumatic manifestation associated with IFN-α therapy. Despite many reports have been anecdotal (38–41), a few studies with large study groups have reported that the frequency of IFN-α-induced SLE ranges from 0.15% (10) to 0.7% (42). By contrast, none of the systemic ADs exhibited a higher risk in the IBT group than in the non-IBT group in our study.

With Regard to a mechanistic perspective, IFN-α is characterized by increased numbers of circulating autoreactive B and T cells (43). IFN-α therapy can tilt the usually tightly controlled balance toward the activation of these autoreactive cells through a vast array of mechanisms (7). Genetic susceptibility factors determine the type of autoimmunity to be developed. The expression of numerous target genes in antigen-presenting cells (APCs) is induced by IFN-α. As a consequence, stimulated APCs enhance promote isotype switching, potently activate autoreactive T cells (44) and humoral autoimmunity. In addition, T-cell autoreactivity by directly promoting T-cell activation and keeping activated T cells alive can be synergistically amplified by IFN-α (7). Type I IFN genes confer dominant disease resistance and trigger autoimmunity in genetically susceptible host (7).

Although our results demonstrated that the standard interferon-based treatment is associated with an increase of the immune-mediated thyroid damage. Autoimmune thyroid diseases are also common in HCV infected patients (45). The HCV is one of the most important viruses associated with autoimmune diseases. HCV may interfere with the mechanisms of self-recognition and functions both on thyroid cells and the immune system (45), where HCV may mimic the structure of some components of thyroid gland or directly destroy thyroid tissue, starting the autoimmune disease. In fact, the lymphoid tissue is a site for the persistence of the infection and chronic immune stimulus, HCV has a significant lymphotropism (46, 47, 48). The chronic stimulation results in: anti-apoptotic effects, autoantibody production, increased cytokine and chemokine secretion and drive for autoimmunity (45).

The strengths of our study include long-term assessment of concurrent ADs, large validation cohort and the large sample size. However, some limitations of our study should be addressed. First, the possibility of misclassification or miscoding cannot be completely ruled out, although the Bureau of NHI randomly and routinely checks patient charts to ensure the quality of claims from all medical institutions. Second, the relationship between the severity of ADs and disease activity in IBT-treated patients with HCV infection could not be analyzed. Third, there is still a possibility of unmeasurable bias given the observational nature of this study, although we used many methods to avoid potential confounders. Finally, some clinical and laboratory data were not available in the administrative database. Additional studies are needed to investigate this association.

In conclusion, our findings revealed that IBT in patients with HCV infection may increase the risk of autoimmune thyroid diseases (Graves’ disease and Hashimoto’s thyroiditis). Therefore, the development of ADs, particularly Graves’ disease and Hashimoto’s thyroiditis, must be monitored in patients receiving IBT. Further mechanistic research should also be conducted.

## Data availability statement

The original contributions presented in the study are included in the article/supplementary material. Further inquiries can be directed to the corresponding author.

## Ethics statement

This study was reviewed and approved by Taipei Medical University Institutional Review Board (Approval Number N201908055). Written informed consent for participation was not required for this study in accordance with the national legislation and the institutional requirements.

## Author contributions

S-MC and H-JY designed research, wrote paper and final approval of the submitted version. T-ML, Y-SC, H-CH, Y-CS, T-TK and S-CC contributed to the data analysis, and final approval of the submitted version. J-HC and C-CC were responsible for the study conception and design, critical article revision for crucial intellectual content, and correspondence regarding the final approval of the submitted version. All authors contributed to the article and approved the submitted version.

## Acknowledgments

This manuscript was edited by Wallace Academic Editing.

## Conflict of interest

The authors declare that the research was conducted in the absence of any commercial or financial relationships that could be construed as a potential conflict of interest.

## Publisher’s note

All claims expressed in this article are solely those of the authors and do not necessarily represent those of their affiliated organizations, or those of the publisher, the editors and the reviewers. Any product that may be evaluated in this article, or claim that may be made by its manufacturer, is not guaranteed or endorsed by the publisher.
